# Crystal Structure of *de Novo* Designed Coiled-Coil Protein Origami Triangle

**DOI:** 10.1021/jacs.3c05531

**Published:** 2023-07-24

**Authors:** Tadej Satler, San Hadži, Roman Jerala

**Affiliations:** † Department of Synthetic Biology and Immunology, National Institute of Chemistry, 1000 Ljubljana, Slovenia; ‡ Interdisciplinary Doctoral Programme in Biomedicine, University of Ljubljana, 1000 Ljubljana, Slovenia; § Department of Physical Chemistry, Faculty of Chemistry and Chemical Technology, University of Ljubljana, 1000 Ljubljana, Slovenia; ∥ EN-FIST Centre of Excellence, 1000 Ljubljana, Slovenia

## Abstract

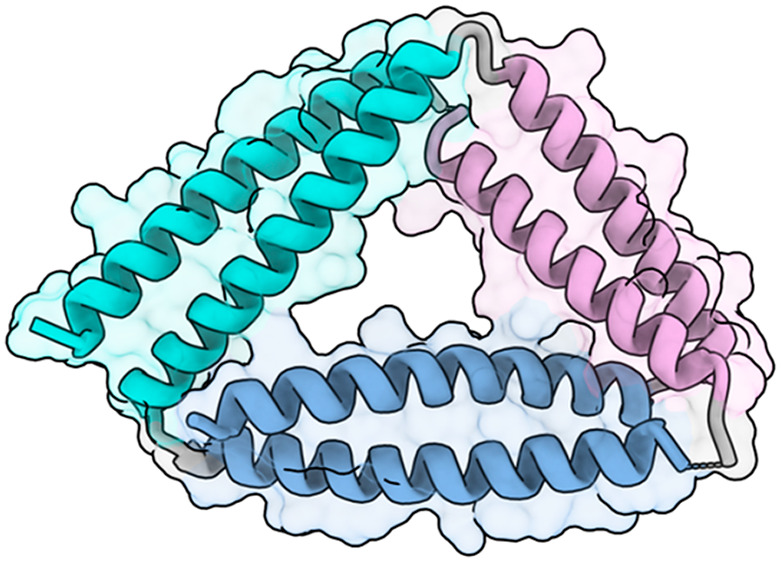

Coiled-coil protein origami (CCPO) uses modular coiled-coil building blocks and topological principles to design polyhedral structures distinct from those of natural globular proteins. While the CCPO strategy has proven successful in designing diverse protein topologies, no high-resolution structural information has been available about these novel protein folds. Here we report the crystal structure of a single-chain CCPO in the shape of a triangle. While neither cyclization nor the addition of nanobodies enabled crystallization, it was ultimately facilitated by the inclusion of a GCN_2_ homodimer. Triangle edges are formed by the orthogonal parallel coiled-coil dimers P1:P2, P3:P4, and GCN_2_ connected by short linkers. A triangle has a large central cavity and is additionally stabilized by side-chain interactions between neighboring segments at each vertex. The crystal lattice is densely packed and stabilized by a large number of contacts between triangles. Interestingly, the polypeptide chain folds into a trefoil-type protein knot topology, and AlphaFold2 fails to predict the correct fold. The structure validates the modular CC-based protein design strategy, providing molecular insight underlying CCPO stabilization and new opportunities for the design.

Recent developments in protein design combined with machine learning enable the design of *de novo* globular proteins.^1−4^ Nevertheless, extensive experimental validation is still required to identify the sequences with desired structure and function.^5^ An alternative strategy to protein scaffold design is to use modular building blocks with a well-understood sequence–structure relationship.^5−9^ In a similar manner, DNA nanotechnology takes advantage of the modular base-pairing in the DNA duplex to design DNA nanostructures.^10,11^ The principle of modular pairing also applies to some protein motifs such as coiled-coils.^12−14^ Coiled-coils (CCs) associate according to well-defined pairing rules encoded in the heptad repeat pattern of *abcdefg* positions.^13,15^ CC dimers pair with high specificity through a combination of hydrophobic and electrostatic interactions at the heptad positions *a*/*d* and *e*/*g*, respectively (Figure 1a).^16^ In the coiled-coil protein origami (CCPO) design strategy, CCs are used as modular building blocks to design protein nanostructures.^17^ The desired shape is defined through the topological arrangement of parallel and/or antiparallel CC dimers arranged into a precisely defined sequential order, based on the underlying mathematical rules.^18,19^ Protein folds such as tetrahedron, bipyramid, as well as multichain assemblies have been assembled using CCPO,^19−22^ and even the folding pathway of those assemblies has been designed.^23^ Although the CCPO has proven to be a robust strategy for the design of various protein topologies and their shape has been confirmed by electron microscopy and small-angle X-ray scattering (SAXS), no high-resolution structural information has been available for these structures. The main difficulty concerns the high flexibility and small size of CCPO structures, which makes them challenging to study using high-resolution methods such as cryoelectron microscopy and X-ray crystallography.

**Figure 1:**
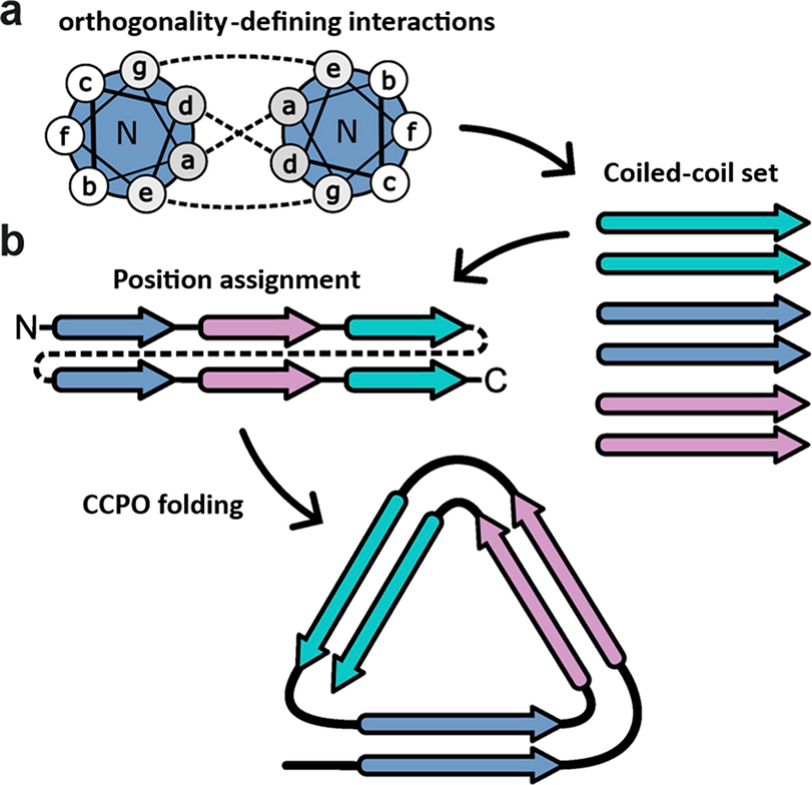
Design of triangular CCPO using coiled-coil building blocks. (a) Helical wheel representation of a parallel coiled-coil with hydrophobic interactions between *a*/*d* residues and electrostatic interactions between *e*/*g* pairs. (b) To design triangular origami, three parallel coiled-coil pairs are selected and arranged sequentially in the polypeptide chain.

To address this issue, we sought to determine the crystal structure of the most elementary CCPO, the triangle. We designed a triangular protein using three orthogonal CC heterodimers concatenated in a single polypeptide chain. The triangular topology can only be achieved using parallel CC dimers since the polypeptide chain has to transverse each triangle edge in the same direction (Figure 1b).

For the initial design, we used charged CC variants (abbreviated SN) P1:P2, P3:P4, and P5:P6^19^ connected with a 5-residue linker (GSGPG) (Table S1). TRI-6SN had CD spectra with high helical content (Figure S1); however, no crystals could be obtained under the studied conditions. As TRI-6SN likely resists crystallization due to flexibility, we designed three variants with shorter linkers, having 1–3 residues (G, GS, GSG). Variants with 2 and 3 residue linkers expressed a high helix content and unfolded cooperatively (Figure S1). However, no crystals were obtained.

We speculated that the termini may be responsible for the high flexibility. Therefore, we designed a cyclized variant TRI-cySN where termini were covalently linked using a trans-splicing reaction based on orthogonal split-inteins (Figure S2).^24^ While cyclization significantly increased protein thermal stability, it still did not lead to crystallization. Finally, we resorted to the ultimate strategy for the crystallization of difficult proteins and used nanobodies as crystallization chaperones. We designed the TRI-SHb variant using stabilized and helical peptides (abbreviated SHb) of P1:P2, P3:P4, and P5:P6. Since no specific nanobodies are available to bind these CCs, we applied an epitope transplantation strategy.^25^ By substituting several solvent-exposed residues, we mimicked the helical epitope of the IB3 intrabody, which binds a helical segment of the huntingtin peptide.^26^ In this way, we successfully introduced the IB3 binding site into P1:P2 or both P1:P2 and P5:P6 pairs (Figure S3). However, even the triangle-nanobody complexes failed to yield any crystals.

Previously we characterized a set of specific nanobodies that recognize different CCs of the designed tetrahedron.^27^ During crystallographic experiments, we observed that nanobody complexes with P5:P6 and P7:P8 CC heterodimers were difficult to crystallize compared to the homodimers, like APH_2_, GCN_2_, and BCR_2_. Based on this experience we tested whether a substitution of one CC heterodimer for a parallel homodimer would improve crystallization. We designed the variant TRI-4SHbGCN where P5:P6 is replaced by a GCN_2_ homodimer, and the segments are connected with GSG linkers (Figures 2a, S4). The purified TRI-4SHbGCN (Figure S5) is monodisperse in solution with the molecular weight 24.5 ± 0.2 kDa, in agreement with the theoretical value, and a hydrodynamic radius of 5.5 ± 1.2 nm (Figure 2b,c). CD analysis shows around 90% helix content, exceptional thermal stability with a melting temperature > 85 °C, and protein refolding ability upon cooling (Figure 2d,e). Importantly, the TRI-4SHbGCN variant crystallized in a range of different conditions.

**Figure 2:**
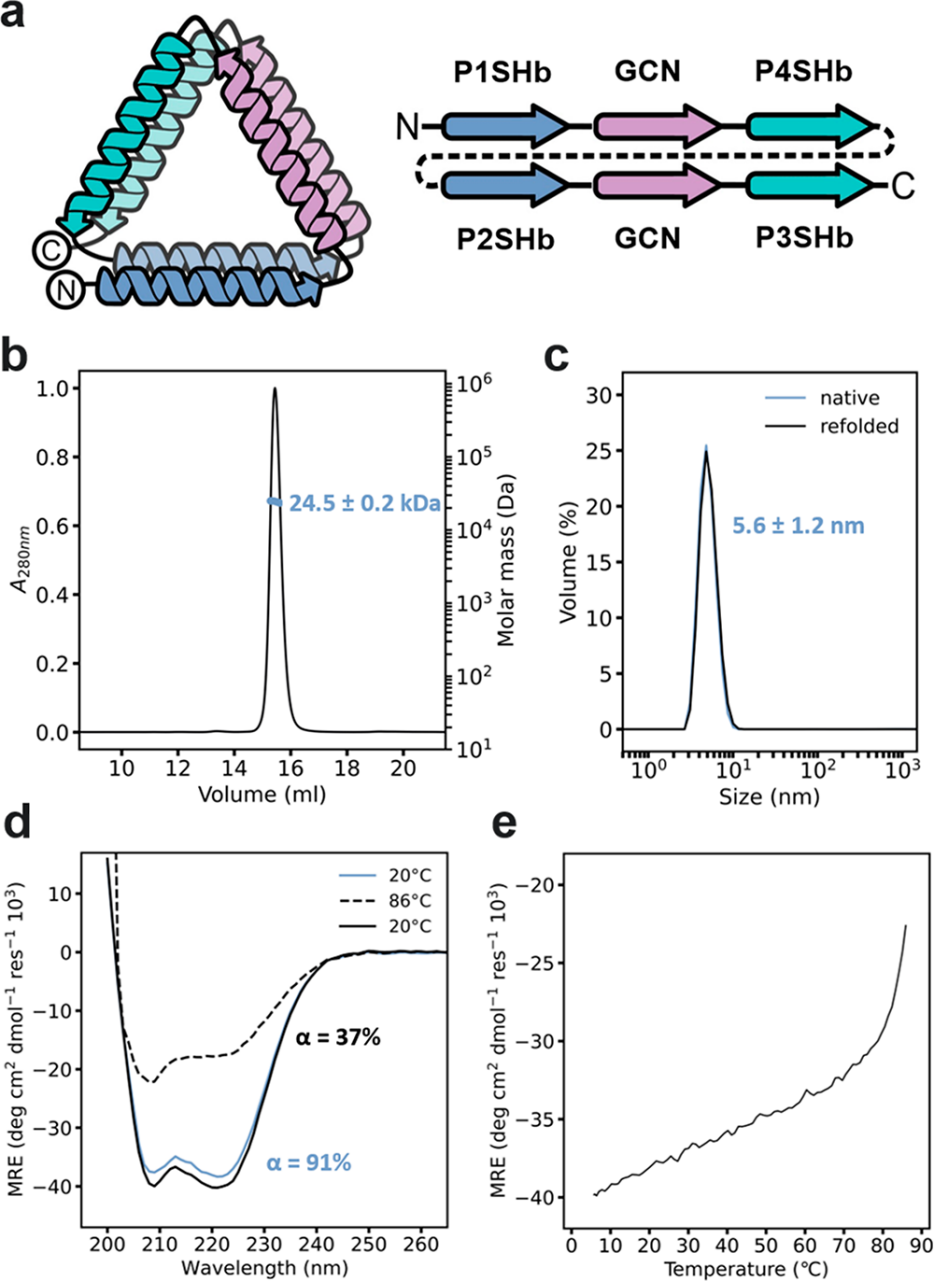
Characterization of TRI-4SHbGCN in solution. (a) Schematic representation of the topological arrangement in TRI-4SHbGCN. (b) Monodispersity and molecular weight analysis, using SEC-MALS. (c) DLS size distribution analysis. (d) CD spectra at 20, 86, and 20 °C postdenaturation. (e) Thermal melting was monitored through CD intensity at 222 nm.

One crystal form belonged to space group *P*1, diffracted to 2.05 Å, and the structure was solved using molecular replacement (PDB: 8P4Y, Table S2). The asymmetric unit contains one TRI-4SHbGCN molecule with a triangular fold as designed (Figure 3a). The triangle is nonequilateral with a shorter GCN_2_ side (34 Å) and two longer (47 Å) P1:P2 and P3:P4 sides. There is an internal cavity of about 600 Å^2^. The electron density is continuous for the entire chain, except for linker sequences, where only two linkers have clear density and could be modeled in the structure. These two linkers are attached to the P4 segment, suggesting this is a more rigid part of the structure, as also reflected in the lower average B-factor for P4 (Figure S6). Interestingly, in the linker connecting P4 to P2 both Ser100 and Gly101 become part of the P2 helix and Ser100 side-chain hydrogen bonds to Trp95 on the preceding P4 segment (Figure 3b). Thus, part of the GSG linker is integrated into the helix, leaving only Gly99 as a flexible linker residue. Our final crystallographic model is consistent with the SAXS profile of the TRI-4SHbGCN in solution (chi^2^ = 1.34) and fits well into the *ab initio* protein envelope calculated from SAXS data (Figure 3c).

**Figure 3:**
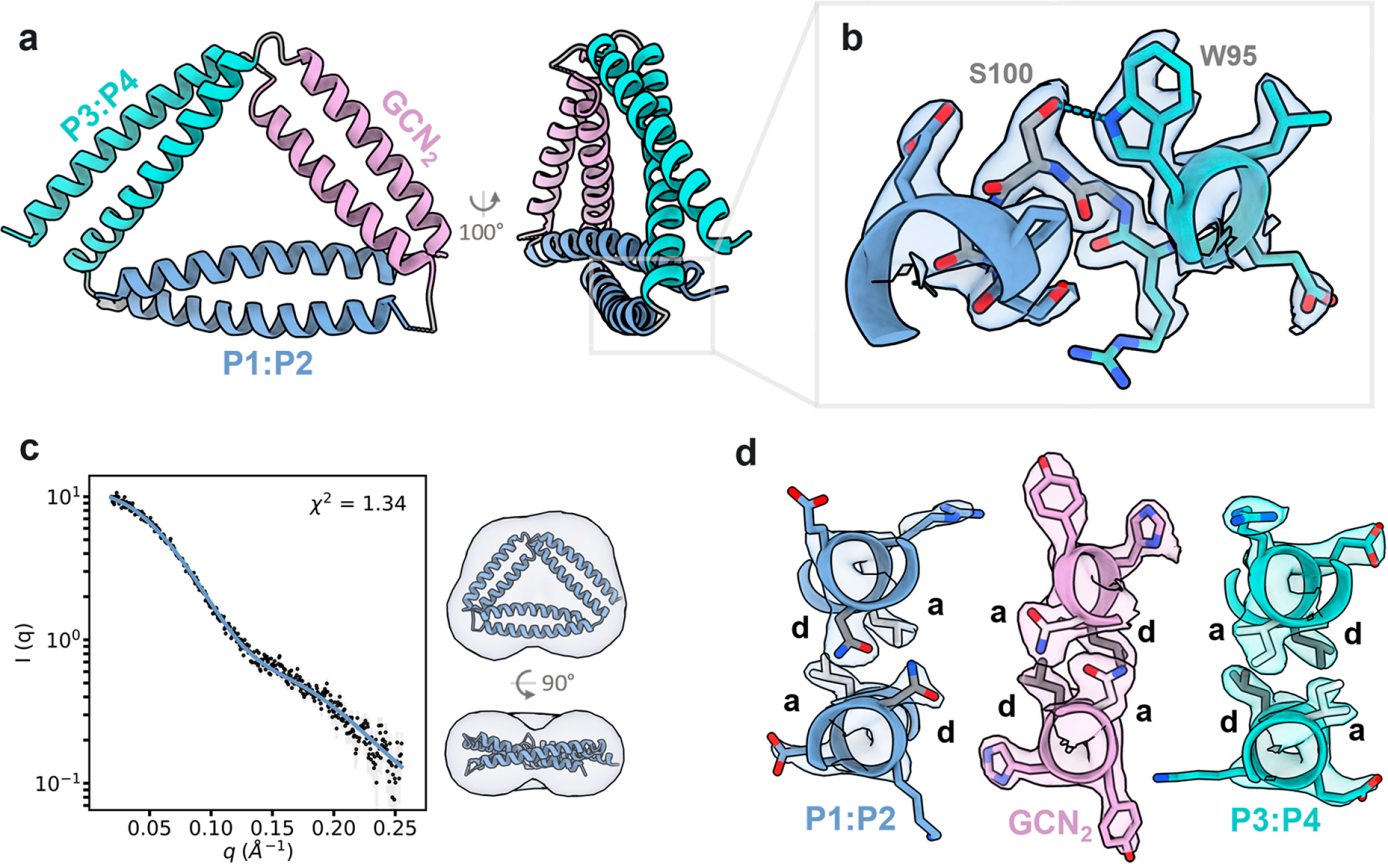
Structure of TRI-4SHbGCN. (a) Crystal structure of TRI-4SHbGCN (PDB: 8P4Y). (b) Structure of a linker visible in the 2*F*_o_–*F*_c_ electron density map. (c) Experimental scattering (black) compared to theoretical SAXS profile calculated from the crystal structure and reconstruction superimposed with the crystal structure. (d) Knobs into holes packing within the individual coiled-coil pairs from the triangular structure.

Individual CC dimers are well resolved in the structure and show the expected packing interactions between *a*/*d* residues (Figure 3d). The CCPO strategy relies on the modularity and orthogonality of CC dimers. It is therefore relevant to examine whether the incorporation of CCs into larger assemblies affects their structure. Superposition of the GCN_2_ dimer as observed in CCPO with the isolated GCN_2_ shows no significant changes in terms of Cα RMSD and Crick’s parameters (Figure S7, Table S3). The structure of P1:P2 and P3:P4 dimers has not been determined before, as the only available structure of CCs from this design set is that of the P5:P6-nanobody complex.^27^ Pairing Asn residues at position *a* has a stabilizing effect and contributes to peptide orthogonality. Within the structure, we observe the formation of a hydrogen bond network involving the backbone on one side and the adjacent Glu and Lys residues on the other side (Figure S8). Superpositions of P1:P2 and P3:P4 with P5:P6 show that the homologous residues at positions *a* and *d* have comparable packing interactions and that the structures are overall similar in terms of Cα RMSD and Crick’s parameters (Figure S7, Table S3). Therefore, the structures of CCs incorporated into the triangular fold remain essentially identical to the structures in isolation.

The crystal lattice is assembled by dense stacking of triangles on top of one another along the *a* and *b* unit cell axis and through end-to-end arrangement along the *c* axis (Figure 4a). Despite the presence of a cavity in the triangle center, the solvent content of crystals is 43.5%, below the average for this point group.^28^

**Figure 4:**
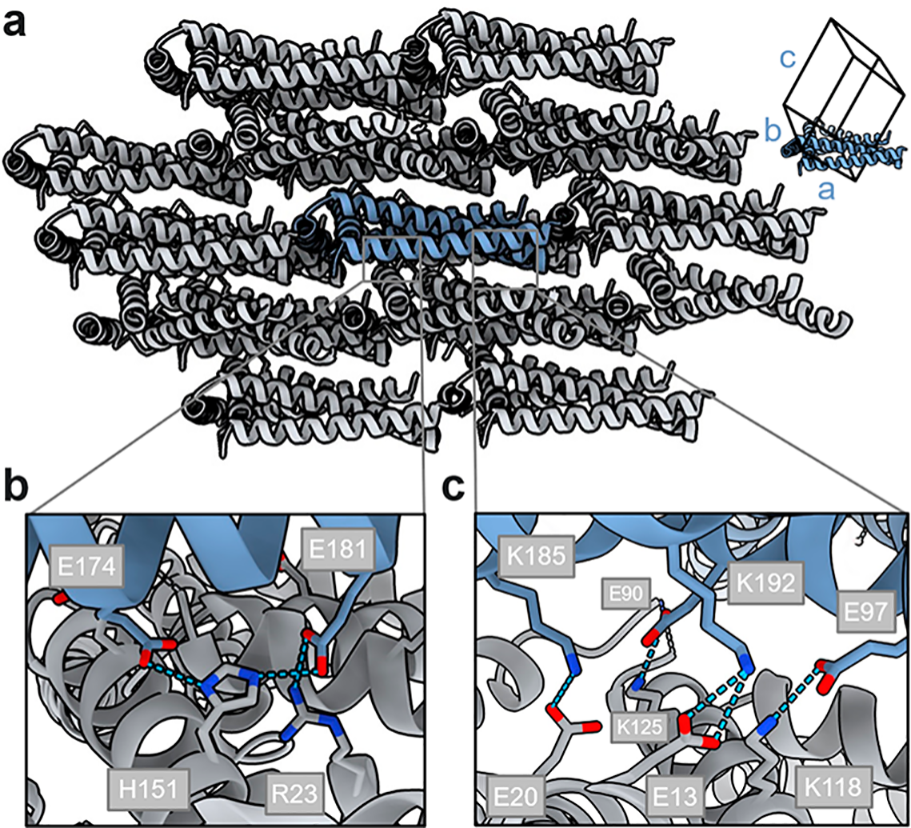
TRI-4SHbGCN crystal lattice and crystal contacts. (a) Triangle molecules densely pack into the crystal lattice. Hydrogen bond network between residues at (b) positions *b*/*c* and *f* and (c) positions *e*/*g*.

Each molecule interacts with 10 symmetry-related molecules via 5 unique interfaces, all of which are heterologous (Figure S9). Crystal packing buries 3600 Å^2^ of solvent-exposed surface area per molecule, which represents about 30% of the molecular surface. While typical crystal contacts are formed via a subset of residues, we observe that a considerable number of the residues are involved in crystal contacts, mostly hydrogen bonds and salt bridges. As expected, the majority of hydrogen bond crystal contacts are formed by the residues at exposed positions *b*/*c* and *f* (Figure 4b); however almost an equal amount of hydrogen bond contacts is established by residues at the *e*/*g* position. Generally, *e*/*g* positions provide electrostatic complementarity between CC dimers, but here, electrostatic interactions between two *e*/*g* positions also promote crystal contacts (Figure 4c).

An unexpected feature of the structure is the interactions between the CC segments at the vertices. The contact map of TRI-4SHbGCN shows the designed interactions between orthogonal CC segments parallel to the main diagonal, while the interactions between CC pairs appear cross-diagonally (Figure 5a). For example, at vertex 1 (P1:P2/GCN_2_) Arg23 at position *c* on the P1 segment stacks against Tyr150 from the second GCN segment. At the neighboring position *f* on P1 Arg26 hydrogen bonds to Asn149 from GCN, while Trp30 packs on top of the Leu-Leu pair of the GCN hydrophobic core (Figure 5b).

**Figure 5:**
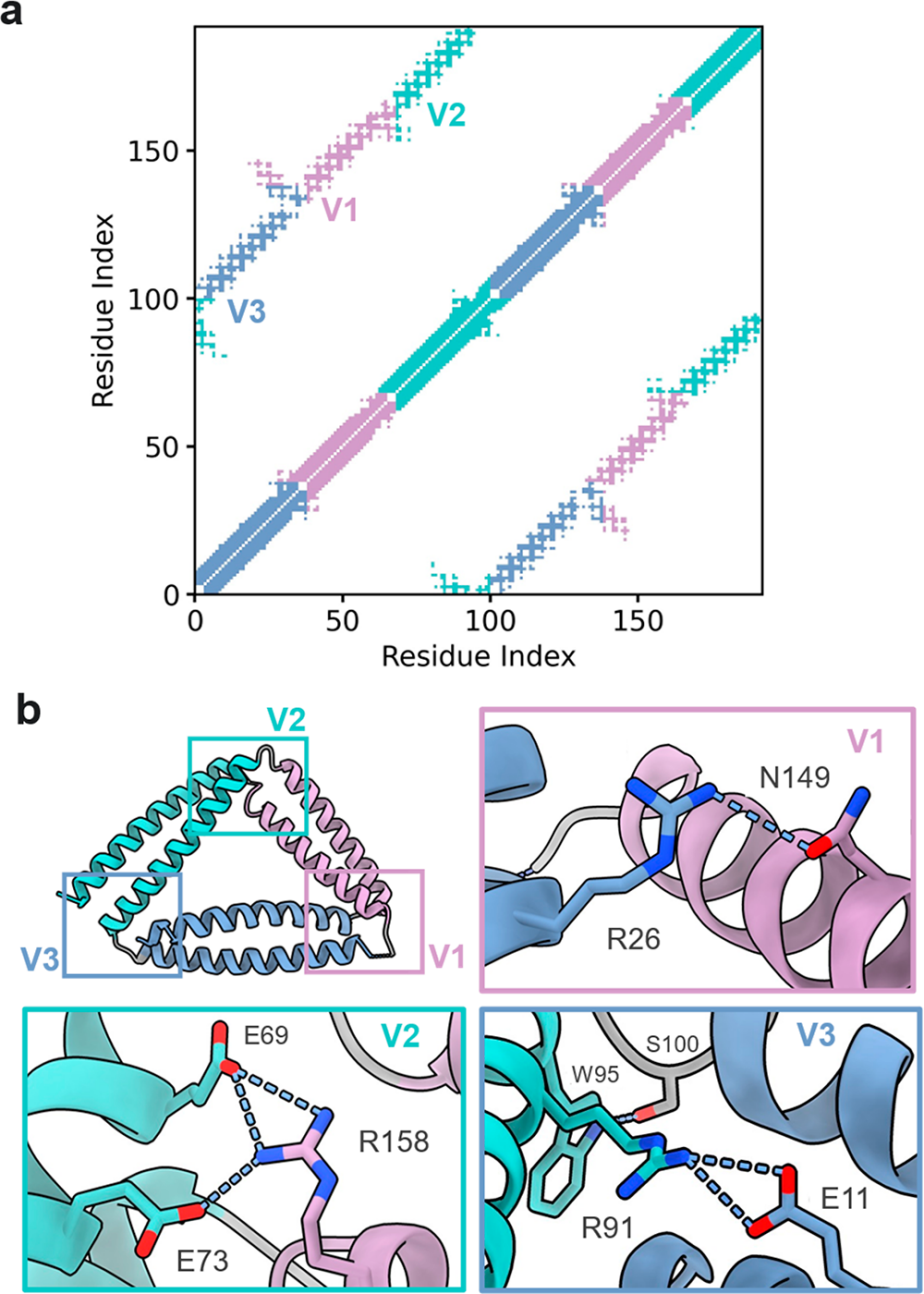
Interactions in TRI-4SHbGCN vertices. (a) TRI-4SHbGCN contact map and (b) close view of hydrogen bond interactions between coiled-coils at vertex 1 (P1:P2-GCN_2_), vertex 2 (P3:P4-GCN_2_), and vertex 3 (P3:P4–P1:P2).

At vertex 2 (GCN_2_/P3:P4) there is a hydrogen bond network between Arg158 from the second GCN segment position *c* and two glutamate side chains (Glu69 and Glu73) on the P4 (Figure 5b). At vertex 3 (P3:P4/P1:P2) Arg91 from P4 forms electrostatic interactions with Glu11 from P1 and P4 Trp95 hydrogen bonds to Ser100 from the GSG linker (Figures 3d, 5b). Although the interactions between CC dimers were not intentionally designed, they likely form due to the acute angles at the triangle vertices, which bring the side chains from the CC segments into proximity.

A closer inspection of the TRI-4SHbGCN topology revealed that the chain forms a relatively shallow protein knot, known as the trefoil-type knot.^29,30^ From the top view, helix segments in each dimer are approximately parallel and alternate by packing on either the inner or outer side of the triangle (Figure S10). From the side view, the helical axis in each dimer is crossing the plane of the triangle (Figure S10) so the first three helix segments are arranged in a triangle that intertwines with the triangle formed by the last three segments. Interestingly, AlphaFold2^31,32^ is unable to predict the fold of TRI-4SHbGCN (and other CCPO structures, Figure S11), most likely due to the complex folding topology and absence of this type of fold in structural databases. CoCoPOD^19^ was used to generate an ensemble of TRI-4SHbGCN models. The knot is not present in these models, and the agreement with SAXS data is systematically worse compared to the crystallographic model (Figure S12). However, due to the SAXS resolution limit, we cannot conclusively resolve whether the knot is present also in the solution.

The presented high-resolution structure not only validates the designed CCPO topology but also reveals previously unobserved structural features such as stabilizing interactions between CC segments at vertices and integration of linkers into the CC helix while also confirming that the structure of CC dimers is unperturbed in the context of protein origami. TRI-4SHbGCN forms, to our knowledge, the smallest knot in a designed protein, occurring due to a supercoil of CCs, similar to designed knots in DNA nanostructures.^33,34^ These findings advance our understanding of the molecular mechanisms underlying CCPO folding and stability and contribute to the development of new coiled-coil-based and knotted synthetic assemblies.

## Notes

TRI-4SHbGCN coordinates have been deposited in the Protein Data Bank under the code 8P4Y.

## ABBREVIATIONS

CC: coiled-coil
CCPO: coiled-coil protein origami
CD: circular dichroism

## References

[ref1] Jumper J.; Evans R.; Pritzel A.; Green T.; Figurnov M.; Ronneberger O.; Tunyasuvunakool K.; Bates R.; Žídek A.; Potapenko A.; Bridgland A.; Meyer C.; Kohl S. A. A.; Ballard A. J.; Cowie A.; Romera-Paredes B.; Nikolov S.; Jain R.; Adler J.; Back T.; Petersen S.; Reiman D.; Clancy E.; Zielinski M.; Steinegger M.; Pacholska M.; Berghammer T.; Bodenstein S.; Silver D.; Vinyals O.; Senior A. W.; Kavukcuoglu K.; Kohli P.; Hassabis D. Highly Accurate Protein Structure Prediction with AlphaFold. Nature 2021 596:7873 2021, 596 (7873), 583–589. 10.1038/s41586-021-03819-2. PMC8371605 34265844

[ref2] Huang P. S.; Boyken S. E.; Baker D. The Coming of Age of de Novo Protein Design. Nature 2016, 537 (7620), 320–327. 10.1038/nature19946. 27629638

[ref3] Wang J.; Lisanza S.; Juergens D.; Tischer D.; Watson J. L.; Castro K. M.; Ragotte R.; Saragovi A.; Milles L. F.; Baek M.; Anishchenko I.; Yang W.; Hicks D. R.; Expòsit M.; Schlichthaerle T.; Chun J. H.; Dauparas J.; Bennett N.; Wicky B. I. M.; Muenks A.; DiMaio F.; Correia B.; Ovchinnikov S.; Baker D. Scaffolding Protein Functional Sites Using Deep Learning. Science (1979) 2022, 377 (6604), 387–394. 10.1126/science.abn210. PMC9621694 35862514

[ref4] Baek M.; DiMaio F.; Anishchenko I.; Dauparas J.; Ovchinnikov S.; Lee G. R.; Wang J.; Cong Q.; Kinch L. N.; Dustin Schaeffer R.; Millán C.; Park H.; Adams C.; Glassman C. R.; DeGiovanni A.; Pereira J. H.; Rodrigues A. V.; Van Dijk A. A.; Ebrecht A. C.; Opperman D. J.; Sagmeister T.; Buhlheller C.; Pavkov-Keller T.; Rathinaswamy M. K.; Dalwadi U.; Yip C. K.; Burke J. E.; Christopher Garcia K.; Grishin N. V.; Adams P. D.; Read R. J.; Baker D. Accurate Prediction of Protein Structures and Interactions Using a Three-Track Neural Network. Science (1979) 2021, 373 (6557), 871–876. 10.1126/science.abj8754. PMC7612213 34282049

[ref5] Broom A.; Trainor K.; MacKenzie D. W. S.; Meiering E. M. Using Natural Sequences and Modularity to Design Common and Novel Protein Topologies. Curr. Opin Struct Biol. 2016, 38, 26–36. 10.1016/j.sbi.2016.05.007. 27270240

[ref6] Parmeggiani F.; Huang P. S. Designing Repeat Proteins: A Modular Approach to Protein Design. Curr. Opin Struct Biol. 2017, 45, 116–123. 10.1016/j.sbi.2017.02.001. 28267654

[ref7] Divine R.; Dang H. V.; Ueda G.; Fallas J. A.; Vulovic I.; Sheffler W.; Saini S.; Zhao Y. T.; Raj I. X.; Morawski P. A.; Jennewein M. F.; Homad L. J.; Wan Y. H.; Tooley M. R.; Seeger F.; Etemadi A.; Fahning M. L.; Lazarovits J.; Roederer A.; Walls A. C.; Stewart L.; Mazloomi M.; King N. P.; Campbell D. J.; McGuire A. T.; Stamatatos L.; Ruohola-Baker H.; Mathieu J.; Veesler D.; Baker D. Designed Proteins Assemble Antibodies into Modular Nanocages. Science (1979) 2021, 372 ( (6537), ), 10.1126/science.abd9994. PMC8592034 33795432

[ref8] Woolfson D. N. A Brief History of De Novo Protein Design: Minimal, Rational, and Computational. J. Mol. Biol. 2021, 433 ( (20), ), 167160 10.1016/j.jmb.2021.167160. 34298061

[ref9] Korendovych I. V.; DeGrado W. F. De Novo Protein Design, a Retrospective. Q. Rev. Biophys. 2020, 53, e3 10.1017/S0033583519000131. 32041676 PMC7243446

[ref10] Seeman N. C.; Sleiman H. F. DNA Nanotechnology. Nature Reviews Materials 2017 3:1 2018, 3 (1), 1–23. 10.1038/natrevmats.2017.68.

[ref11] Wagenbauer K. F.; Sigl C.; Dietz H. Gigadalton-Scale Shape-Programmable DNA Assemblies. Nature 2017 552:7683 2017, 552 (7683), 78–83. 10.1038/nature24651. 29219966

[ref12] Lapenta F.; Aupič J.; Strmšek Z.; Jerala R. Coiled Coil Protein Origami: From Modular Design Principles towards Biotechnological Applications. Chem. Soc. Rev. 2018, 47 (10), 3530–3542. 10.1039/C7CS00822H. 29400389

[ref13] Woolfson D. N. Understanding a Protein Fold: The Physics, Chemistry, and Biology of α-Helical Coiled Coils. J. Biol. Chem. 2023, 299 (4), 104579 10.1016/j.jbc.2023.104579. 36871758 PMC10124910

[ref14] Park W. M.; Bedewy M.; Berggren K. K.; Keating A. E. Modular Assembly of a Protein Nanotriangle Using Orthogonally Interacting Coiled Coils. Scientific Reports 2017 7:1 2017, 7 (1), 1–10. 10.1038/s41598-017-10918-6. PMC5585338 28874805

[ref15] Lupas A. N.; Gruber M. The Structure of α-Helical Coiled Coils. Adv. Protein Chem. 2005, 70, 37–38. 10.1016/S0065-3233(05)70003-6. 15837513

[ref16] Woolfson D. N. Coiled-Coil Design: Updated and Upgraded. Subcell Biochem 2017, 82, 35–61. 10.1007/978-3-319-49674-0_2. 28101858

[ref17] Gradišar H.; Božič S.; Doles T.; Vengust D.; Hafner-Bratkovič I.; Mertelj A.; Webb B.; Šali A.; Klavžar S.; Jerala R. Design of a Single-Chain Polypeptide Tetrahedron Assembled from Coiled-Coil Segments. Nature Chemical Biology 2013 9:6 2013, 9 (6), 362–366. 10.1038/nchembio.1248. PMC3661711 23624438

[ref18] Gradišar H.; Božič S.; Doles T.; Vengust D.; Hafner-Bratkovič I.; Mertelj A.; Webb B.; Šali A.; Klavžar S.; Jerala R. Design of a Single-Chain Polypeptide Tetrahedron Assembled from Coiled-Coil Segments. Nature Chemical Biology 2013 9:6 2013, 9 (6), 362–366. 10.1038/nchembio.1248. PMC3661711 23624438

[ref19] Ljubetič A.; Lapenta F.; Gradišar H.; Drobnak I.; Aupič J.; Strmšek Z.; Lainšček D.; Hafner-Bratkovič I.; Majerle A.; Krivec N.; Benčina M.; Pisanski T.; Veličković T. Ć.; Round A.; Carazo J. M.; Melero R.; Jerala R. Design of Coiled-Coil Protein-Origami Cages That Self-Assemble in Vitro and in Vivo. Nat. Biotechnol. 2017, 35 (11), 1094–1101. 10.1038/nbt.3994. 29035374

[ref20] Božic S.; Abram S.; Gradišar H.; Aupič J.; Round A. R.; Jerala R. Triangular in Vivo Self-Assembling Coiled-Coil Protein Origami. ACS Chem. Biol. 2021, 16 (2), 310–315. 10.1021/acschembio.0c00812. 33476117 PMC7901019

[ref21] Aupič J.; Lapenta F.; Strmšek Z.; Merljak E.; Plaper T.; Jerala R. Metal Ion-Regulated Assembly of Designed Modular Protein Cages. Sci. Adv. 2022, 8 (24), 8243 10.1126/sciadv.abm8243. PMC9205593 35714197

[ref22] Lapenta F.; Aupič J.; Vezzoli M.; Strmšek Z.; da Vela S.; Svergun D. I.; Carazo J. M.; Melero R.; Jerala R. Self-Assembly and Regulation of Protein Cages from Pre-Organised Coiled-Coil Modules. Nat. Commun. 2021, 12 (1), 1–12. 10.1038/s41467-021-21184-6. 33574245 PMC7878516

[ref23] Aupič J.; Strmšek Z.; Lapenta F.; Pahovnik D.; Pisanski T.; Drobnak I.; Ljubetič A.; Jerala R. Designed Folding Pathway of Modular Coiled-Coil-Based Proteins. Nat. Commun. 2021, 12 (1), 1–12. 10.1038/s41467-021-21185-5. 33574262 PMC7878764

[ref24] Carvajal-Vallejos P.; Pallissé R.; Mootz H. D.; Schmidt S. R. Unprecedented Rates and Efficiencies Revealed for New Natural Split Inteins from Metagenomic Sources. J. Biol. Chem. 2012, 287 (34), 28686–28696. 10.1074/jbc.M112.372680. 22753413 PMC3436554

[ref25] Kim J. W.; Kim S.; Lee H.; Cho G.; Kim S. C.; Lee H.; Jin M. S.; Lee J. O. Application of Antihelix Antibodies in Protein Structure Determination. Proc. Natl. Acad. Sci. U. S. A. 2019, 116 (36), 17786–17791. 10.1073/pnas.1910080116. 31371498 PMC6731670

[ref26] Schiefner A.; Chatwell L.; Körner J.; Neumaier I.; Colby D. W.; Volkmer R.; Wittrup K. D.; Skerra A. A Disulfide-Free Single-Domain V(L) Intrabody with Blocking Activity towards Huntingtin Reveals a Novel Mode of Epitope Recognition. J. Mol. Biol. 2011, 414 (3), 337–355. 10.1016/j.jmb.2011.09.034. 21968397

[ref27] Majerle A.; Hadzi S.; Aupič J.; Satler T.; Lapenta F.; Strmšek Z.; Lah J.; Loris R.; Jerala R. A Nanobody Toolbox Targeting Dimeric Coiled-Coil Modules for Functionalization of Designed Protein Origami Structures. Proc. Natl. Acad. Sci. U. S. A. 2021, 118 ( (17), ), 10.1073/pnas.2021899118. PMC8092592 33893235

[ref28] Chruszcz M.; Potrzebowski W.; Zimmerman M. D.; Grabowski M.; Zheng H.; Lasota P.; Minor W. Analysis of Solvent Content and Oligomeric States in Protein Crystals—Does Symmetry Matter?. Protein Sci. 2008, 17 (4), 623 10.1110/ps.073360508. 18359856 PMC2271157

[ref29] Kolesov G.; Virnau P.; Kardar M.; Mirny L. A. Protein Knot Server: Detection of Knots in Protein Structures. Nucleic Acids Res. 2007, 35, 10.1093/NAR/GKM312. PMC1933242 17517776

[ref30] Virnau P.; Mirny L. A.; Kardar M. Intricate Knots in Proteins: Function and Evolution. PLoS Comput. Biol. 2006, 2 (9), 1074–1079. 10.1371/journal.pcbi.0020122. PMC1570178 16978047

[ref31] Mirdita M.; Schütze K.; Moriwaki Y.; Heo L.; Ovchinnikov S.; Steinegger M. ColabFold: Making Protein Folding Accessible to All. Nature Methods 2022 19:6 2022, 19 (6), 679–682. 10.1038/s41592-022-01488-1. PMC9184281 35637307

[ref32] Evans R.; O’Neill M.; Pritzel A. Protein Complex Prediction with AlphaFold-Multimer. bioRxiv. 2022, 10.1101/2021.10.04.463034 (accessed 2023–05–20).

[ref33] Kočar V.; Schreck J. S.; Čeru S.; Gradišar H.; Bašić N.; Pisanski T.; Doye J. P. K.; Jerala R. Design Principles for Rapid Folding of Knotted DNA Nanostructures. Nat. Commun. 2016, 7, 10.1038/ncomms10803. PMC4759626 26887681

[ref34] Scalvini B.; Sheikhhassani V.; Woodard J.; Aupič J.; Dame R. T.; Jerala R.; Mashaghi A. Topology of Folded Molecular Chains: From Single Biomolecules to Engineered Origami. Trends Chem. 2020, 2 (7), 609–622. 10.1016/j.trechm.2020.04.009.

