# Supplementary material for: Crystal Structure of de Novo Designed Coiled-Coil Protein Origami Triangle
Source: J Am Chem Soc. 2023 Jul 24;145(31):16995–7000. doi: 10.1021/jacs.3c05531 (PMC10416210; doi:10.1021/jacs.3c05531)
Supplement: Supplementary file 1 — ja3c05531_si_001.pdf [file ja3c05531_si_001.pdf]

Supplementary Information

Crystal Structure of De Novo Designed Coiled-Coil Protein Origami Triangle

Tadej Satler[†,‡], San Hadži[†,§*], and Roman Jerala[†,‖*]

† Department of Synthetic Biology and Immunology, National Institute of Chemistry, Ljubljana, Slovenia

‡ Interdisciplinary Doctoral Programme in Biomedicine, University of Ljubljana, Ljubljana, Slovenia.

§ Department of Physical Chemistry, Faculty of Chemistry and Chemical Technology, University of Ljubljana, Ljubljana, Slovenia

‖ EN-FIST Centre of Excellence, Ljubljana, Slovenia.

*Corresponding authors.

This document includes:

- Materials and Methods
- Supplementary Figures 1 to 12
- Supplementary Tables 1 to 3
- Supplementary References

Materials and Methods

Molecular cloning. *E. Coli* strain DH5-α was used to perform molecular cloning experiments using the Gibson assembly protocol [1]. Synthetic genes were ordered from Twist Bioscience and had complementary overhangs to the pET-41a(+) vector, that was previously linearized with PCR amplification. The ligation was achieved by incubating synthetic genes and the target vector for 30 minutes at 50°C in the reaction mixture described in [1]. Next, the reaction mixture was used to transform *E. Coli* using the heat-shock method. Positive clones and the DNA sequences were confirmed using colony PCR and Sanger sequencing.

Protein production. Prepared TEV protease, IB3 intrabody, and CCPO protein constructs were transformed into *E. Coli* strain NiCo21(DE3) and grown overnight on kanamycin-supplemented LB agar plates at 37°C. A single colony was then used to prepare the inoculum and grow it overnight in LB medium supplemented with kanamycin. The inoculums were then added to 5-liter fermentation flasks containing 1 liter of LB media to reach an optical density (OD) of 0.1 and grown at 37°C until OD values reached between 0.6 and 0.8. The cultures were then induced with 0.5 mM Isopropyl β-D-1-thiogalactopyranoside (IPTG) and grown overnight at 22°C. Afterward, bacterial pellets were harvested by centrifugation and stored at -80°C.

Protein purification. The isolation protocol for TEV protease, IB3 intrabody, and CCPO proteins was composed of Ni-NTA affinity and size-exclusion chromatography. Frozen pellets were resuspended on ice in the lysis buffer (50mM Tris-HCl pH 8.0, 150mM NaCl, 10mM imidazole, Benzonase, and CPI protease inhibitor mix), lysed by sonication (about 7 minutes of effective sonication in intervals of 1 s ON and 3 s OFF with 55% amplitude) and centrifuged at 16000 x g for 20 min at 4°C. Supernatant or soluble fraction was filtered through a 0.45-µm syringe filter and applied to the Ni-NTA resin in a gravity column, previously equilibrated with buffer A (50mM Tris-HCl pH 8.0, 150mM NaCl, 10mM imidazole). After extensive washing with buffer A and buffer B (50mM Tris-HCl pH 8.0, 150mM NaCl, 20mM imidazole), the sample was eluted with buffer C (50mM Tris-HCl pH 8.0, 150mM NaCl, 300mM imidazole) and fractions containing the protein of interest were filtered with 0.22-µm syringe filters and injected to size-exclusion HiLoad 26/600 Superdex 200 pg column, previously equilibrated with Tris buffer (20 mM Tris-HCl pH 7.5, 150 mM NaCl). Chromatography was performed using an AKTA pure FPLC system with a 2.6 ml/min linear flow rate. Eluted protein fractions were analyzed with SDS-PAGE (sodium dodecyl sulfate polyacrylamide gel electrophoresis).

Intein reaction (protein cyclization). The CCPO construct pairs with orthogonal split-inteins were isolated separately with Ni-NTA affinity and size-exclusion chromatography. To perform the trans-splicing reaction, the protein pairs were mixed at a final concentration of 5-20 µM in Tris-buffer supplemented with 1-2M Urea and 5mM TCEP, and incubated overnight at 37°C. To remove unwanted by-products that contained a His-tag or that were too large, the mixture was passed through Ni-NTA resin and then re-injected onto a size-exclusion column. The eluted cyclized product was analyzed with SDS-PAGE.

TEV protease cleavage. TEV protease was purified using Ni-NTA and size-exclusion chromatography methods as described in the protocol above. TEV protease was used for the

cleavage of the His-tag from the CCPO constructs. Controlled proteolysis was performed by adding TEV protease to the target protein (~50-100 molar excess of target protein). The mixture was supplemented with 1 mM TCEP and then incubated for 2 hours at room temperature, followed by overnight incubation at 4°C. To clear the sample of uncleaved products, cleaved His-tags, and TEV protease, the mixture was passed through Ni-NTA resin. The eluted sample was collected for further analysis.

Protein electrophoresis. Proteins were analyzed with SDS-PAGE in 12 or 15 % discontinuous polyacrylamide gels. Samples in loading buffer and pre-stained ruler for molecular weight were run on the gel for 55 min at 200 V. The proteins were then visualized by staining the gel with the InstantBlue agent.

Circular dichroism (CD) spectrometry. Samples were prepared in Tris buffer (20 mM Tris-HCl pH 7.5, 150 mM NaCl) with a protein concentration between 0.3 mg/ml and 0.5 mg/ml. The experiments were conducted on a Chirascan CD spectrometer equipped with a Peltier temperature controller. CD spectra were recorded in the far-UV region, from 200 to 280 nm, with a step size of 1 nm, a bandwidth of 1 nm, and 1 second sampling. All the measurements were performed in 8 replicates and subsequently averaged. The helical content of the proteins was calculated from the following equation [2].

$$Helical\ content\ (\%) = 100\ x\ \frac{MRE_{222} - MRE_{coil}}{MRE_{helix}\ x\ \left(1 - \frac{3}{n}\right) - MRE_{coil}} \quad (1)$$

In the equation for calculating helical content from MRE at 222 nm, n is the length of the amino acid sequence, MRE_{coil} = 640-45T, and MRE_{helix} is the theoretical mean residue ellipticity of an infinitely long helix (−42,500 deg cm^2 $dmol^{-1}$).
Thermal denaturation experiments were carried out in a temperature gradient from 5°C to 90°C, at a heating rate of 1°C/min. The CD signal was measured at 222 nm at 1°C increments throughout the experiment.

Dynamic light scattering (DLS). The size distribution of particles was recorded on a Zetasizer Nano at 20°C. Samples were in Tris buffer (20 mM Tris-HCl pH 7.5, 150 mM NaCl) with a protein concentration between 0.3 mg/ml and 0.5 mg/ml. The hydrodynamic diameter was calculated with software provided by the manufacturer.

Size-exclusion chromatography coupled to multi-angle light scattering (SEC-MALS). SEC-MALS measurements were performed using a Waters e2695 HPLC system coupled with a UV detector, multiple-angle light scattering detector Dawn8+, and a refractive index (RI) detector RI500. The flow rate used during the chromatography was 0.5 mL/min. Protein samples were filtered through 0.1-µm centrifuge filters and then injected onto a Superdex 200 Increase 10/300 column, which was previously equilibrated with Tris buffer (20 mM Tris-HCl at pH 7.5 and 150 mM NaCl). Data analysis was done using Astra 7.0 software.

Small-angle X-ray scattering (SAXS). Samples were prepared in Tris buffer (20 mM Tris-HCl pH 7.5, 150 mM NaCl) supplemented with 10% glycerol. Measurements were performed

at 10°C on an Anton Paar SAXSpoint 5.0 equipped with Primux 100 micro Cu X-ray source, 2D 1M EIGER2 R series detector, and ASX autosampler. 2D scattering patterns at various sample concentrations (for TRI-4SHbGCN - 26.5, 13.3, 6.7, 3.3, and 1.2 mg/ml) and their matching buffers were collected as 7 frames of 30 min exposure per frame with a distance between sample and detector of 600 mm. After manual inspection for radiation damage and a buffer mismatch, 1D raw SAXS curves were calculated from the appropriate samples using SAXS analysis software. Additional manipulation of the SAXS curves was then performed using the ATSAS software package [3]. Scattering profiles were analyzed using the PRIMUS software [4], Ab initio shape determination was performed with the DAMMIF program [5] and the SAXS profile of our protein model was calculated and compared with experimental data using Pepsi-SAXS [6].

Isothermal titration calorimetry (ITC). All the samples were dialyzed three times against a phosphate buffer (20 mM sodium phosphate buffer pH 6.8 and 150 mM NaCl), centrifuged for 10 minutes, and degassed. The titrations were performed with a MicroCal VP-ITC instrument at 25°C. Solutions containing binders were titrated into the sample cell containing 5-10 µM of CCPO triangles, with the binder solution being about ten times more concentrated. Raw thermograms were integrated with the software NITPIC [7], interaction analysis was done with SEDPHAT [8], and titration curves were visualized with a companion GUSSI software.

Crystallization and structure determination. Triangular proteins and protein complexes with nanobodies were prepared in Tris buffer (20 mM Tris-HCl pH 7.5, 150 mM NaCl) at concentrations between 7-15 mg/ml and dispensed to crystallization plates using Phoenix robot from Art Robbins. Protein complexes were prepared by mixing triangular protein with 1.1 molar excess of the corresponding nanobody. Crystallization trials were set as a sitting drops of 0.2 µl of protein solution and 0.2 µl of reservoir solution from different commercial crystallization screens (PACT premier, JCSG Plus, Morpheus, BCS screen, Wizard Cryo 1&2, SG1 screen, HELIX screen).

TRI-4SHbGCN protein was plated at a concentration of 8 mg/ml and after a few months, well-diffracting crystals grew in a solution of 100 mM CHES/NaOH pH 9.5, 200 mM NaCl and 50% (v/v) PEG 400. The crystals were flash-frozen in liquid nitrogen without additional cryoprotectants. X-ray diffraction data was collected at 100K on a beamline PROXIMA1 using a PILATUS 6 M detector. All data were indexed, integrated, and scaled with XDS [9]. Poly-alanine protein model of GCN coiled-coil peptide (PDB 7A4T) was used to solve structure with molecular replacement using PHASER-MR [10]. After phasing initial structural model was built with Buccaneer [11], which was then used in manual rebuilding using COOT [12] and iterative refinement using Refmac 5 [13] and phenix.refine [14]. The final refinement cycles included TLS refinement with 1 helix per TLS group.

Crystal structure analysis. UCSF Chimera [15] and PISA server [16] were used to analyze protein interactions, crystal contacts and to perform a structural comparison. An online knot server was used to analyze the protein backbone [17], while Socket2 [18] and Twister [19] were utilized to determine the structural parameters and knobs into holes residue packing of individual coiled-coil pairs.

Supplementary figures

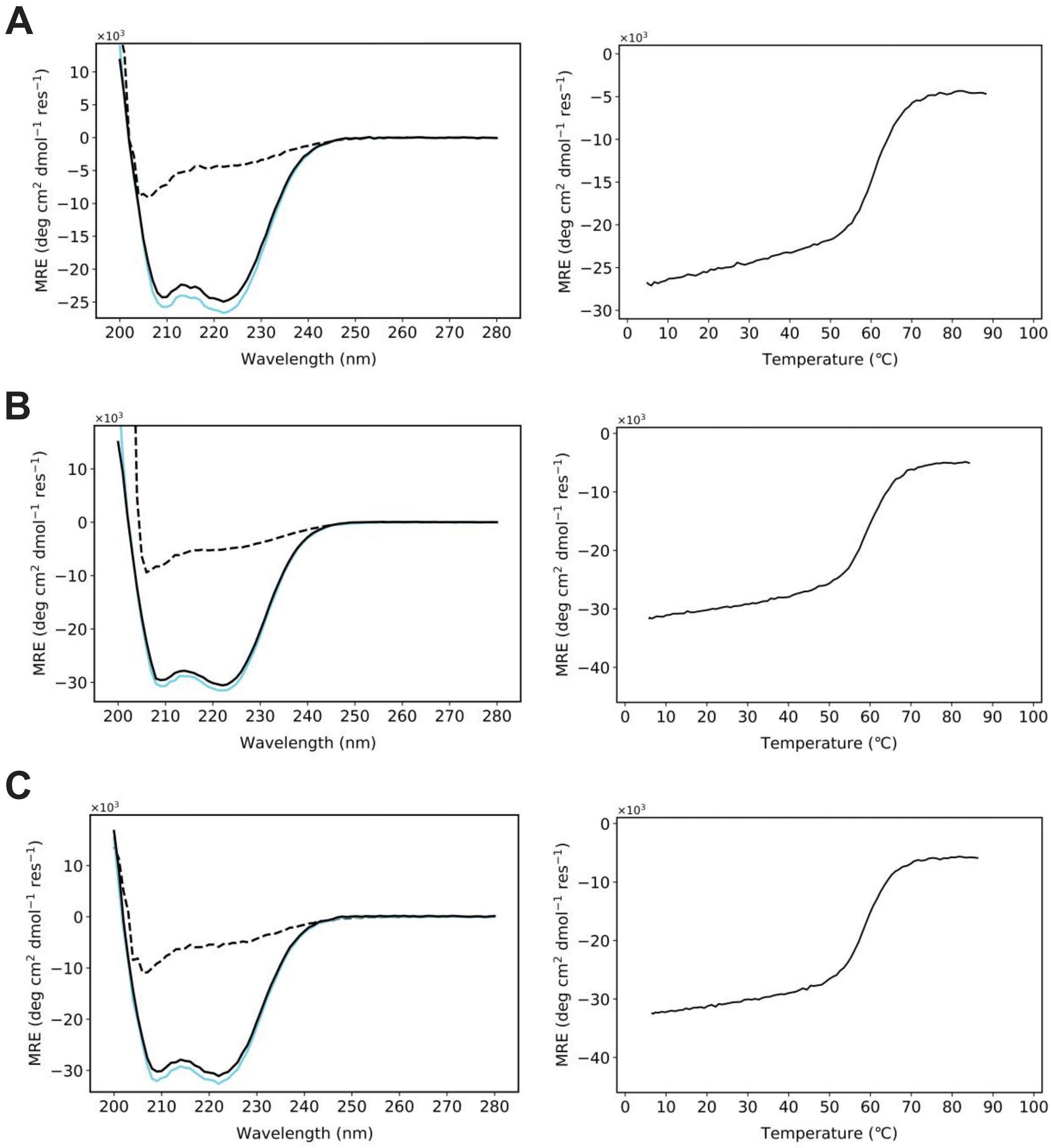

Figure S1. Analysis of TRI-6SN variants with CD. CD spectra and thermal denaturation profile of TRI-6SN variants with (A) GSGPG, (B) GSG, and (C) GS linkers. CD spectra were measured at 20°C, 86°C, and 20°C again after thermal denaturation.

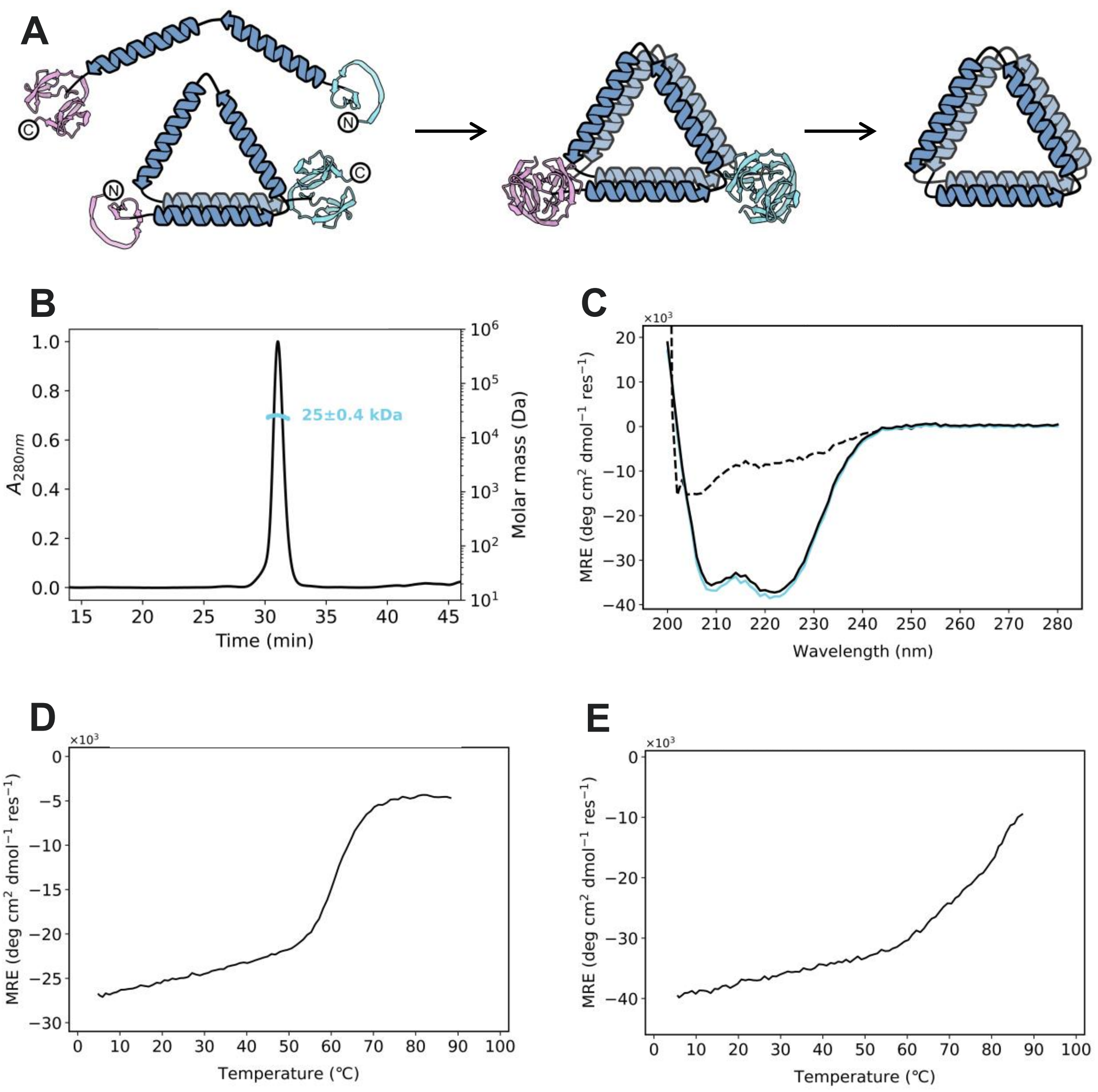

Figure S2. Analysis of cyclized triangular CCPO. (A) Schematic representation of protein cyclization with trans-splicing reaction utilizing orthogonal split-inteins. (B) SEC-MALS chromatogram of cyclized protein and molecular weight calculated from light scattering. (C) CD spectra at 20℃, 86℃, and 20℃ again after thermal denaturation. (D) Thermal denaturation profile of uncyclized control protein and (E) cyclized protein.

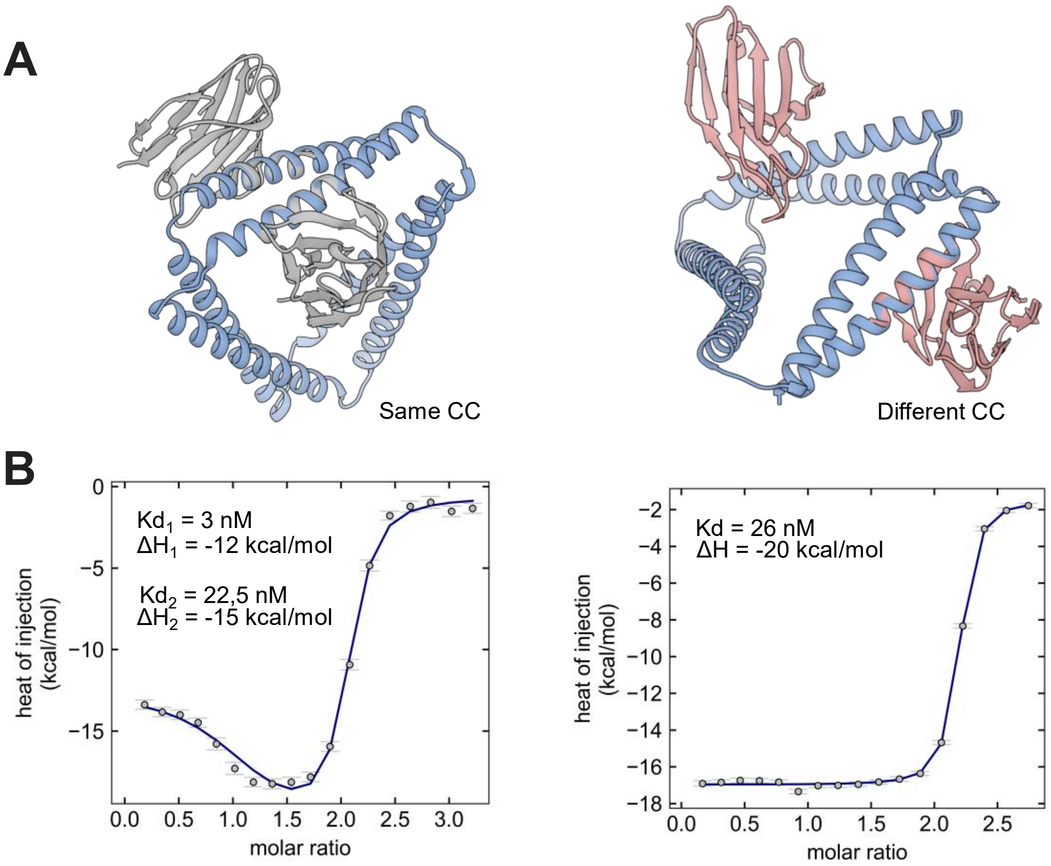

Figure S3. Binding analysis of IB3 to TRI-SHb variants with transplanted helical epitopes. (A) Protein models where two IB3 molecules bind to the same CC (left) and different CC (right). (B) Binding analysis using isothermal titration calorimetry (ITC).

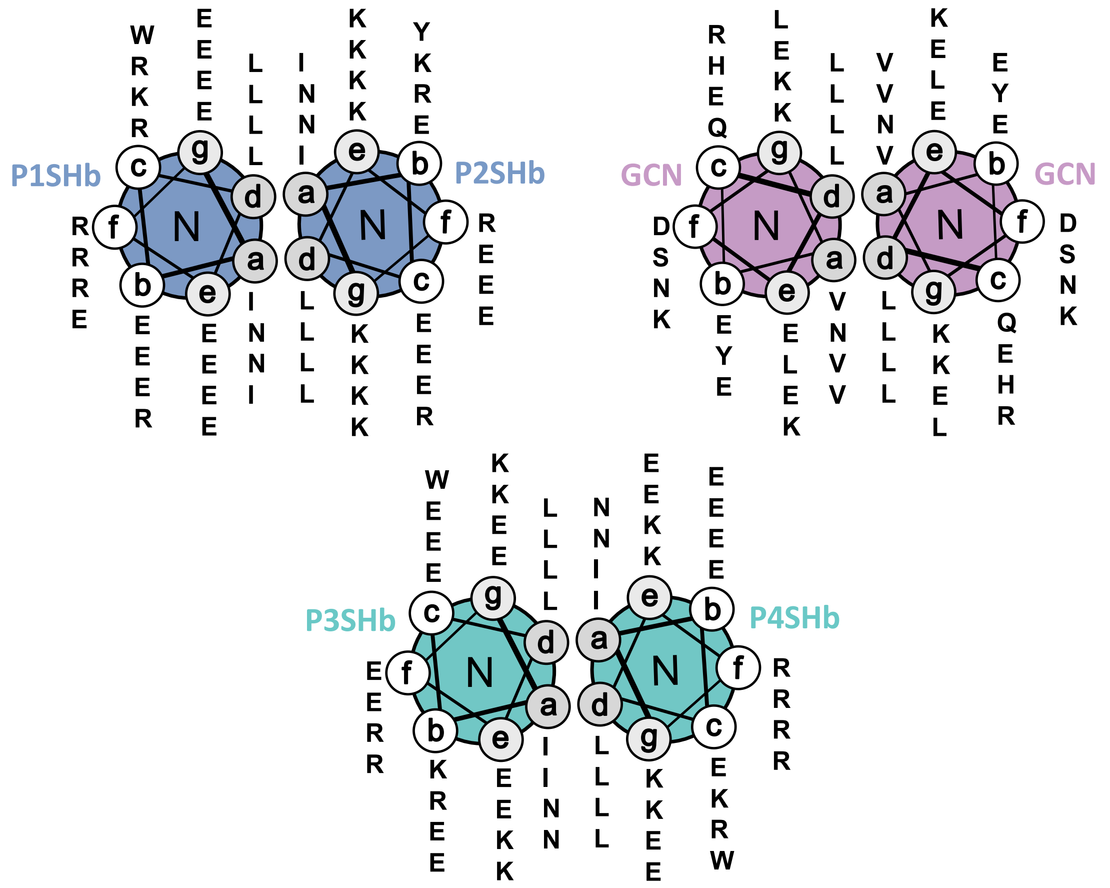

Figure S4. Helical wheel representation of coiled-coil dimers from TRI-4SHbGCN. The view is shown looking down the superhelical axis from N-terminus to C-terminus.

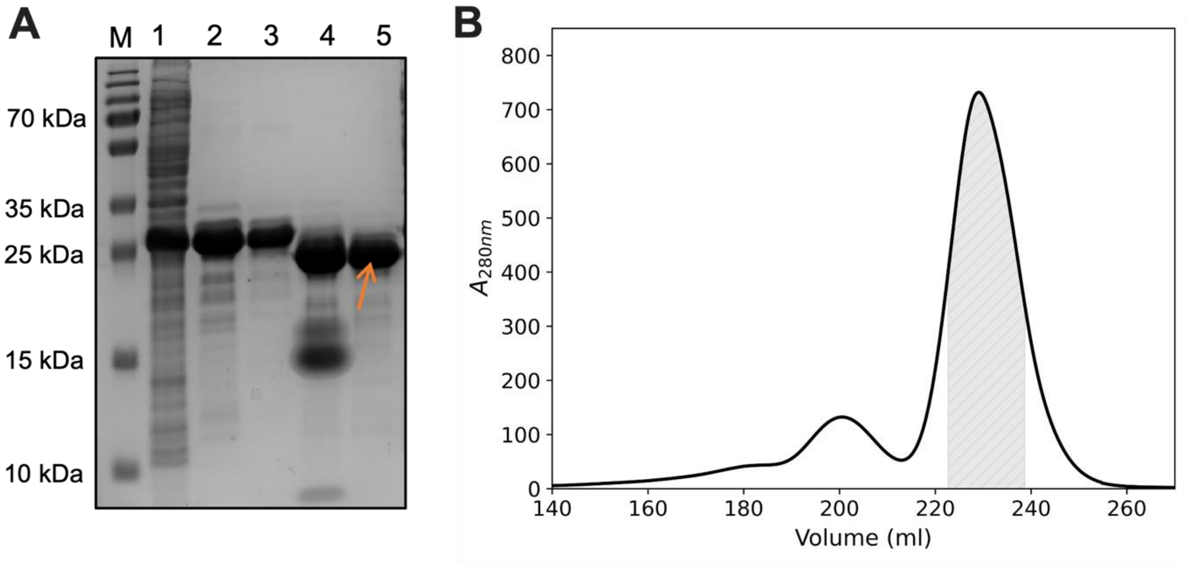

Figure S5. Purification analysis of TRI-4SHbGCN. (A) SDS-PAGE analysis of protein purification. Wells are in the following order: protein marker, soluble fraction, elution from Ni-NTA, protein after size-exclusion chromatography, controlled proteolysis with TEV protease, and final protein after reverse Ni-NTA. (B) Size-exclusion chromatogram of the protein and selected fractions.

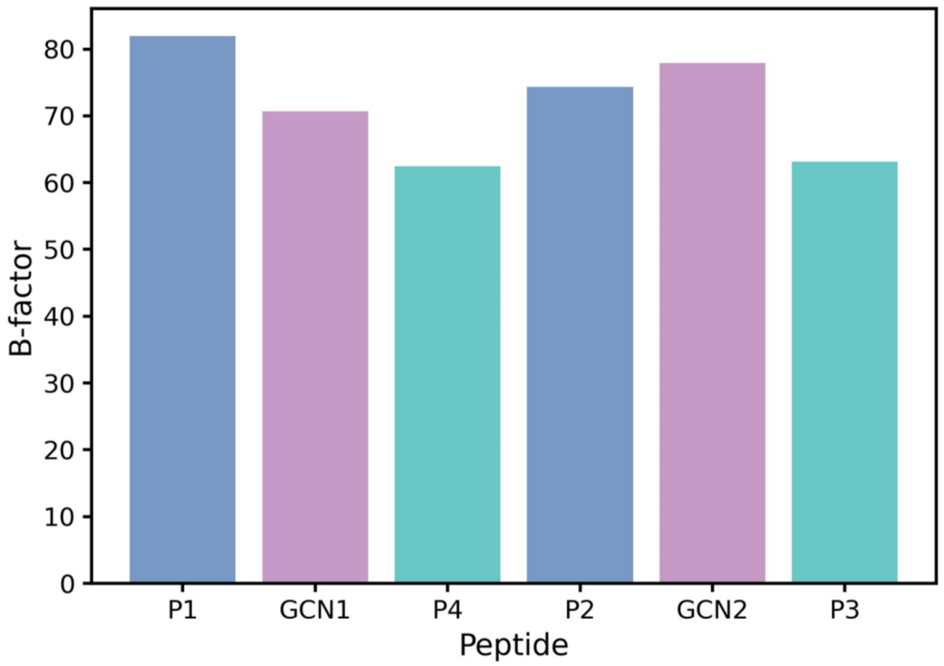

Figure S6. Average Cα B-factor values for individual peptide segments in TRI-4SHbGCN. P3 and P4 have the lowest B-factor values, suggesting they are the most rigid CC pair.

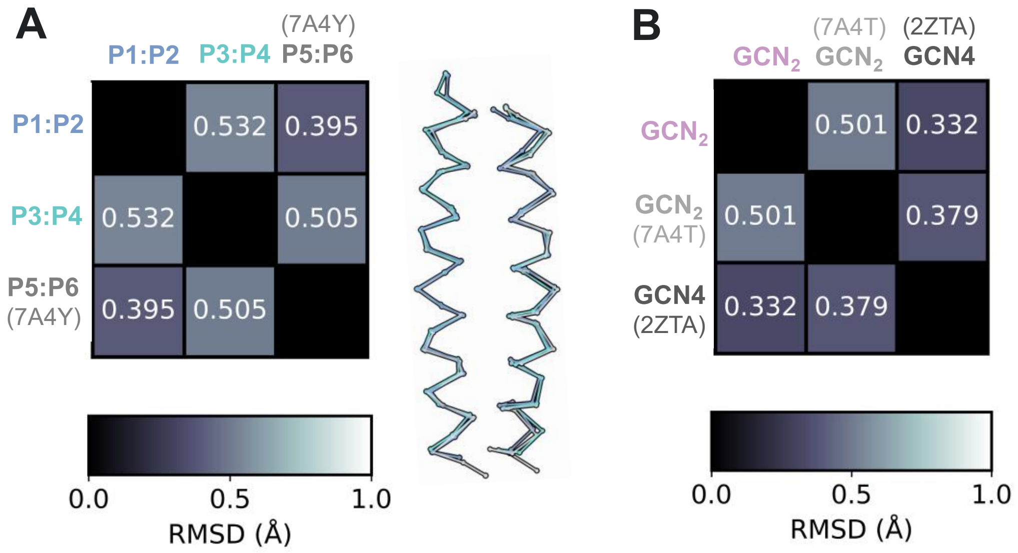

Figure S7. Comparative analysis of backbone RMSD values between isolated CC dimers and CC dimers from TRI-4SHbGCN. (A) Structural superposition of CC dimers P1:P2, P3:P4, together with P5:P6 structure from the complex with nanobody (PDB: 7A4Y). (B) Structural superposition of CC dimer GCN_2 with the structure from the complex with nanobody (PDB: 7A4T) and GCN4 structure (PDB: 2ZTA).

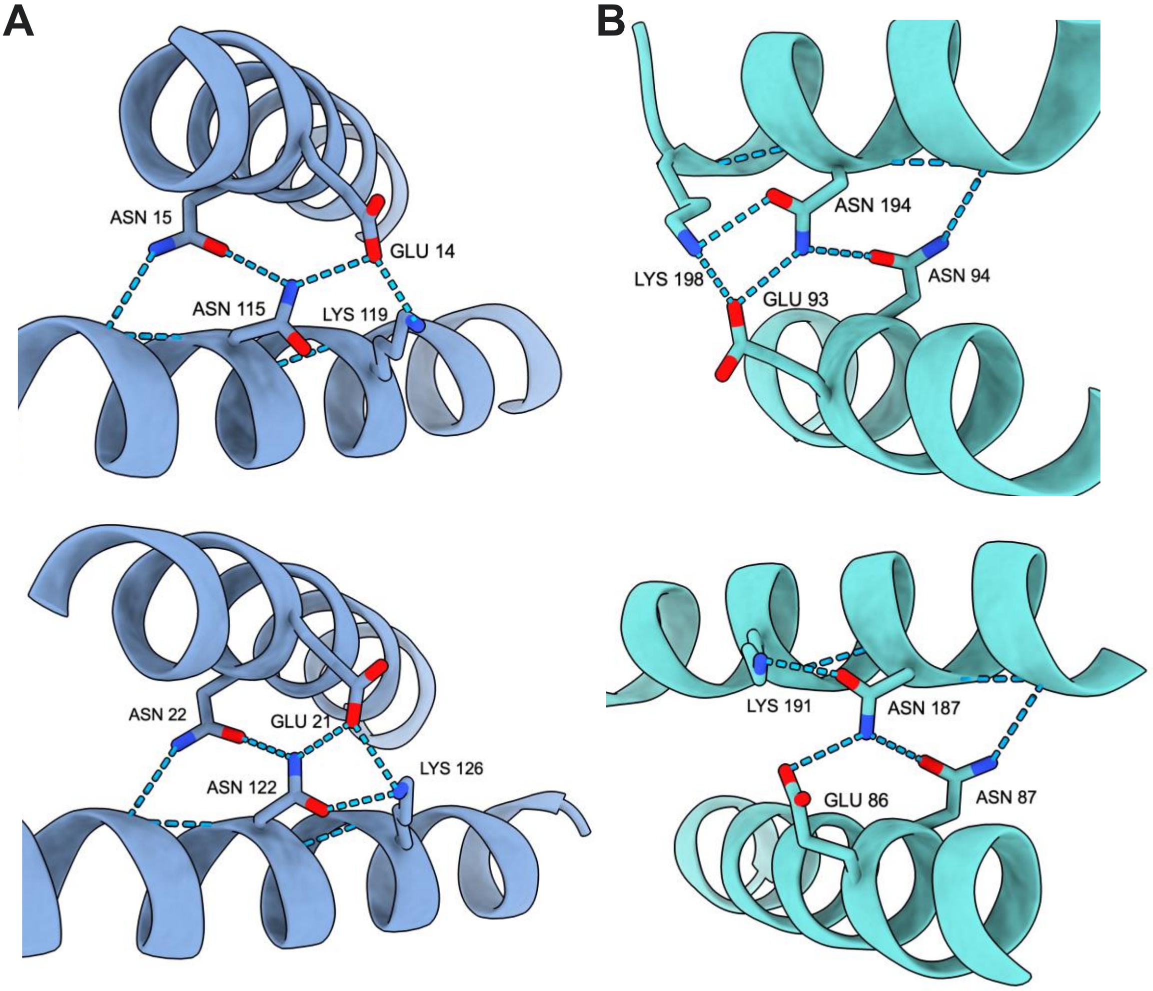

Figure S8. Coiled-coil stabilization via hydrogen bond network. Asn residues at the positions a interacts with a backbone on the one side and adjacent Glu and Lys residues on the other side in (A) P1:P2 and (B) P3:P4 coiled-coil dimers.

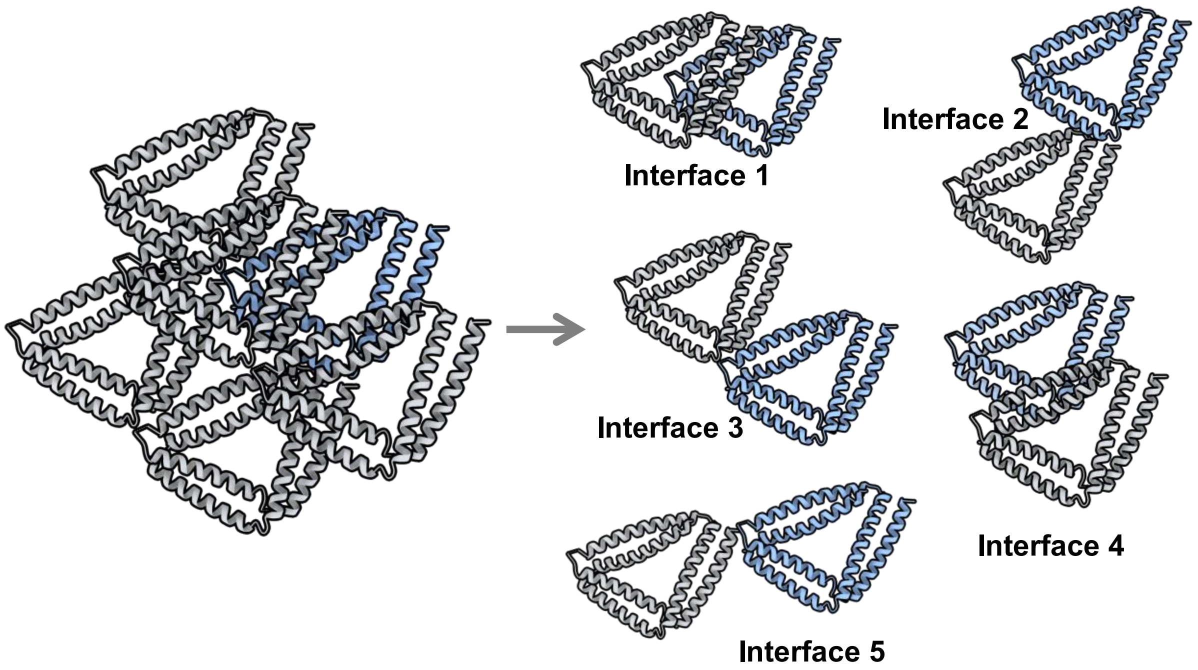

Figure S9. Unique interactions in the crystal lattice of TRI-4SHbGCN. The protein interacts with 10 symmetry-related molecules via 5 unique heterologous interfaces.

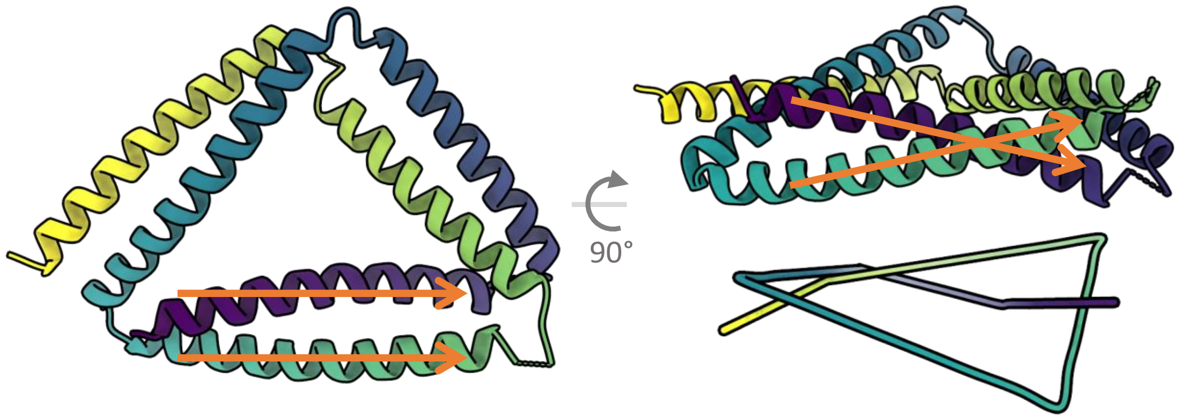

Figure S10. Trefoil knot topology of TRI-4SHbGCN. From the top view (left) helices appear parallel and alternate between the inner and outer sides, while from the side view helices are crossing the plane of the triangle (top right). This resulted in a relatively shallow trefoil protein knot, as depicted from the knot server [17] (bottom right).

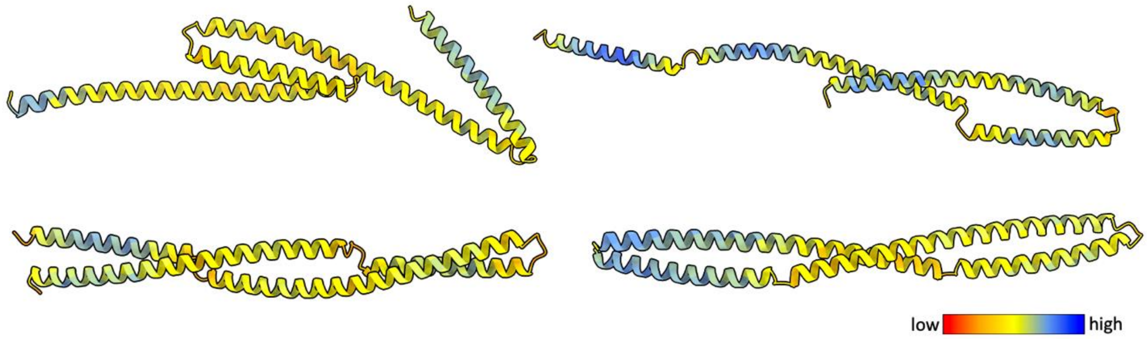

Figure S11. AlphaFold2 models of TRI-4SHbGCN. Shown are a few resulting AF2 protein models colored by pLDDT confidence values. Colabfold was used with the alphafold_multimer_v3 weights either in normal mode or template mode (PDB70). Although the AF2 can generate coiled-coil structures, it has difficulty distinguishing between parallel and antiparallel orientations, which leads to elongated conformations of the protein models instead of the proposed triangular shape.

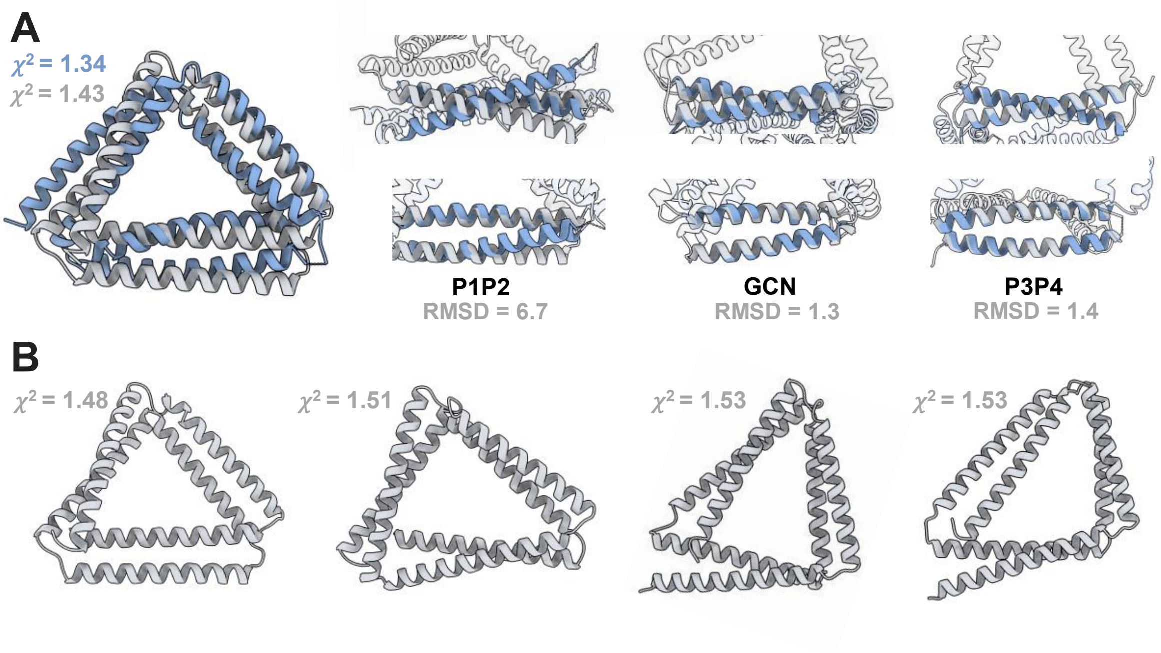

Figure S12. Comparing TRI-4SHBGCN structure with protein models generated with CoCoPOD. (A) Comparison of coiled-coil modules between crystal structure and best protein model (of 300 generated) based on chi-squared ($\chi 2$) scores from SAXS scattering profile. (B) Additional protein model variations with high chi-squared ($\chi 2$) scores.

Supplementary tables

Table S1. Protein sequences.

Protein	Annotation and protein sequence
**TRI-6SN**	**peptide positions: P1SN-P5SN-P4SN-P2SN-P6SN-P3SN**<br>**linker: GSGPG**<br>MGHHHHHHHHENLYFQGSPEDEIRQLEQENSQLERENQRLEQEIYQLERGS<br>GPGSPEDENSQLEEKISQLKQKNSELKEEIQQLEYGSGPGSPEDKISQLKEKIQ<br>QLKQENQQLEEENSQLEYGSGPGSPEDKIEELKEKNSQLKEKNEELKQKIYEL<br>KEGSGPGSPEDKNSELKEEIQQLEEENQQLEEKISELKYGSGPGSPEDEIQQLE<br>EEISQLEQKNSELKEKNQELKY
**cyTRI-6SN (cyclized variant)**	**peptide positions: P1SN-P5SN-P4SN-P2SN-P6SN-P3SN**<br>**linker: GSGPG**<br>SGPGSPEDEIRQLEQENSQLERENQRLEQEIYQLERGSGPGSPEDENSQLEEK<br>ISQLKQKNSELKEEIQQLEYGSGPGSPEDKISQLKEKIQQLKQENQQLEEENS<br>QLEYGSGPGSPEDKIEELKEKNSQLKEKNEELKQKIYELKEGSGPGSPEDKNS<br>ELKEEIQQLEEENQQLEEKISELKYGSGPGSPEDEIQQLEEEISQLEQKNSELKE<br>KNQELKYG
**TRI-6SN (GSG)**	**peptide positions: P1SN-P5SN-P4SN-P2SN-P6SN-P3SN**<br>**linker: GSG**<br>MGHHHHHHHHENLYFQGSPEDEIRQLEQENSQLERENQRLEQEIYQLERGS<br>GSPEDENSQLEEKISQLKQKNSELKEEIQQLEYGSGSPEDKISQLKEKIQQLKQ<br>ENQQLEEENSQLEYGSGSPEDKIEELKEKNSQLKEKNEELKQKIYELKEGSGSP<br>EDKNSELKEEIQQLEEENQQLEEKISELKYGSGSPEDEIQQLEEEISQLEQKNS<br>ELKEKNQELKY
**TRI-6SN (GS)**	**peptide positions: P1SN-P5SN-P4SN-P2SN-P6SN-P3SN**<br>**linker: GS**<br>MGHHHHHHHHENLYFQGSPEDEIRQLEQENSQLERENQRLEQEIYQLERGS<br>SPEDENSQLEEKISQLKQKNSELKEEIQQLEYGSSPEDKISQLKEKIQQLKQEN<br>QQLEEENSQLEYGSSPEDKIEELKEKNSQLKEKNEELKQKIYELKEGSSPEDKN<br>SELKEEIQQLEEENQQLEEKISELKYGSSPEDEIQQLEEEISQLEQKNSELKEKN<br>QELKY
**TRI-6SN (G)**	**peptide positions: P1SN-P5SN-P4SN-P2SN-P6SN-P3SN**<br>**linker: G**<br>MGHHHHHHHHENLYFQGSPEDEIRQLEQENSQLERENQRLEQEIYQLERGS<br>PEDENSQLEEKISQLKQKNSELKEEIQQLEYGSPEDKISQLKEKIQQLKQENQ<br>QLEEENSQLEYGSPEDKIEELKEKNSQLKEKNEELKQKIYELKEGSPEDKNSEL<br>KEEIQQLEEENQQLEEKISELKYGSPEDEIQQLEEEISQLEQKNSELKEKNQEL<br>KY
**TRI-6SHb**	**peptide positions: P1SHb-P5SHb-P4SHb-P2SHb-P6SHb-P3SHb**<br>**linker: GSGPG**

	MGHHHHHHHHENLYFQGSPEDEIERLERENEKLERENERLEREIRWLEEGSG PGSPEDENRELEEKIRELKEKNEELKREIKYLEEGSGPGSPEDKIEELKRKIEKLK RENERLERENEWLERGSGPGSPEDKIEELKRKNRELKEKNKELKEKIYRLKEG SGPGSPEDKNEELKREIERLEEENRELERKIEYLKRGSGPGSPEDEIKELEEEIREL EEKNEELKRKNEWLKR
**TRI-6SHb-14** [1] **(binds 2xIB3)**	**peptide positions: P1SHb-P5SHb-P4SHb-P2SHb-P6SHb-P3SHb** **linker: GSGPG** MGHHHHHHHHENLYFQGSPEDEIKLLEAFNSLLEFENQRLEQEIYQLERGSG PGSPEDENRELEEKIRELKEKNEELKREIKYLEEGSGPGSPEDKIEELKRKIEKLK RENERLERENEWLERGSGPGSPEDKIEELKRKNKLLKAFNSLLKFKIYRLKEGS GPGSPEDKNEELKREIERLEEENRELERKIEYLKRGSGPGSPEDEIKELEEEIREL EEKNEELKRKNEWLKR
**TRI-6SHb-15** [2] **(binds 2xIB3)**	**peptide positions: P1SHb-P5SHb-P4SHb-P2SHb-P6SHb-P3SHb** **linker: GSGPG** MGHHHHHHHHENLYFQGSPEDEIKLLEAFNSLLEFENQRLEQEIYQLERGSG PGSPEDENRELEEKIRELKEKNEELKREIKYLEEGSGPGSPEDKIEELKRKIEKLK RENERLERENEWLERGSGPGSPEDKIEELKRKNRELKEKNKELKEKIYRLKEG SGPGSPEDKNEELKREIKLLEAFNSLLEFKIEYLKRGSGPGSPEDEIKELEEEIRE LEEKNEELKRKNEWLKR
**TRI-4SHbGCN**	**peptide positions: P1SHb-GCN-P4SHb-P2SHb-GCN-P3SHb** **linker: GSG** MGHHHHHHHHENLYFQGSPEDEIERLERENEKLERENERLEREIRWLEEGSG QLEDKVEELLSKNYHLENEVERLKKLVGSGSPEDKIEELKRKIEKLKRENERLE RENEWLERGSGSPEDKIEELKRKNRELKEKNKELKEKIYRLKEGSGQLEDKVE ELLSKNYHLENEVERLKKLVGSGSPEDEIKELEEEIRELEEKNEELKRKNEWLK RGSG
**IB3 (Intrabody)**	MGSQPVLTQSPSVSAAPRQRVTISVSGSNSNIGSNTVNWIQQLPGRAPELLM YDDDLLAPGVSDRFSGSRSGTSASLTISGLQSEDEADYYAATWDDSLNGWVF GGGTKVTVSSHHHHHH

[1] Epitope residues (violet) were transplanted on the same CC dimer
[2] Epitope residues (violet) were transplanted on different CC dimers

Table S2. Data collection and refinement statistics of TRI-4SHbGCN. Statistics for the highest-resolution shell are shown in parentheses.

**PDB code**	8P4Y
**Diffraction source**	SOLEIL synchrotron, Proxima I
**Wavelength (Å)**	0.9786
**Temperature (K)**	100
**Detector**	PILATUS 6M
**Crystal-detector distance (mm)**	296.66
**Rotation range per image (°)**	0.1
**Total rotation range (°)**	360
**Space group**	P1
**a, b, c (Å)**	33.8, 37.0, 44.7
**α, β, γ (°)**	97.3 105.8 95.5
**Mosaicity (°)**	0.169
**Resolution range (Å)**	36.35 - 2.05 (2.13 - 2.05)
**Total No. of reflections**	45207 (7517)
**No. of unique reflections**	12496 (1233)
**Completeness (%)**	97.53 (96.18)
**Redundancy**	3.62 (6.10)
**⟨ I/σ(I)⟩**	15.08 (1.32)
**R_{meas}**	0.039 (0.929)
**CC1/2**	99.9 (68.7)
**Overall B factor from Wilson plot (Å^2)**	58.00
**R-work**	0.2047 (0.3467)
**R-free**	0.2536 (0.3663)
**Protein residues**	192
**RMS(bonds)**	0.007
**RMS(angles)**	0.74
**Ramachandran favored (%)**	100.0
**Ramachandran allowed (%)**	0.0
**Ramachandran outliers (%)**	0.0

**Clash score**	4.56
**Number of non-hydrogen atoms**	1667
**macromolecules**	1631
**ligands**	18
**water**	18
**Average B-factor**	81.5
**Macromolecules**	81.3
**Ligands**	97.6
**Solvent**	77.0

Table S3. Crick's structural parameters of coiled-coils.

Coiled-coil (PDB)	Radius (Å)	Pitch (Å)	Angle (°)
**P1:P2**	5.16 ± 0.07	120.6 ± 7.4	21.27
**P3:P4**	4.94 ± 0.09	152.3 ± 19.9	26.32
**P5:P6 (7A4Y)**	5.12 ± 0.32	119.1 ± 30	20.25
**GCN_2**	4.89 ± 0.17	135.3 ± 25.9	28.49
**GCN_2 (7A4T)**	4.84 ± 0.08	129.7 ± 20	19.82

Supplementary references

[1] D. G. Gibson, L. Young, R.-Y. Chuang, J. C. Venter, C. A. Hutchison, and H. O. Smith, "Enzymatic assembly of DNA molecules up to several hundred kilobases," *Nat Methods*, vol. 6, no. 5, pp. 343–345, May 2009, doi: 10.1038/nmeth.1318.

[2] J. K. Myers, C. N. Pace, and J. M. Scholtz, "A direct comparison of helix propensity in proteins and peptides," *Proc Natl Acad Sci U S A*, vol. 94, no. 7, pp. 2833–2837, Apr. 1997, doi: 10.1073/PNAS.94.7.2833/ASSET/BD108B89-4E20-40EF-A031-6B8F35A012E9/ASSETS/GRAPHIC/PQ0673969003.JPEG.

[3] K. Manalastas-Cantos *et al.*, "ATSAS 3.0: expanded functionality and new tools for small-angle scattering data analysis," *J Appl Crystallogr*, vol. 54, no. Pt 1, pp. 343–355, Feb. 2021, doi: 10.1107/S1600576720013412.

[4] P. v. Konarev, V. v. Volkov, A. v. Sokolova, M. H. J. Koch, and D. I. Svergun, "PRIMUS: a Windows PC-based system for small-angle scattering data analysis," *urn:issn:0021-8898*, vol. 36, no. 5, pp. 1277–1282, Sep. 2003, doi: 10.1107/S0021889803012779.

[5] D. Franke and D. I. Svergun, "DAMMIF, a program for rapid ab-initio shape determination in small-angle scattering," *J Appl Crystallogr*, vol. 42, no. Pt 2, pp. 342–346, 2009, doi: 10.1107/S0021889809000338.

[6] S. Grudinin, M. Garkavenko, and A. Kazennov, "Pepsi-SAXS: an adaptive method for rapid and accurate computation of small-angle X-ray scattering profiles," *Acta Crystallogr D Struct Biol*, vol. 73, no. Pt 5, pp. 449–464, May 2017, doi: 10.1107/S2059798317005745.

[7] S. Keller, C. Vargas, H. Zhao, G. Piszczek, C. A. Brautigam, and P. Schuck, "High-precision isothermal titration calorimetry with automated peak-shape analysis," *Anal Chem*, vol. 84, no. 11, pp. 5066–5073, Jun. 2012, doi: 10.1021/AC3007522/SUPPL_FILE/AC3007522_SI_001.PDF.

[8] H. Zhao, G. Piszczek, and P. Schuck, "SEDPHAT – a platform for global ITC analysis and global multi-method analysis of molecular interactions," *Methods*, vol. 76, p. 137, Apr. 2015, doi: 10.1016/J.YMETH.2014.11.012.

[9] W. Kabsch and IUCr, "XDS," *urn:issn:0907-4449*, vol. 66, no. 2, pp. 125–132, Jan. 2010, doi: 10.1107/S0907444909047337.

[10] A. J. McCoy, R. W. Grosse-Kunstleve, P. D. Adams, M. D. Winn, L. C. Storoni, and R. J. Read, "Phaser crystallographic software," *urn:issn:0021-8898*, vol. 40, no. 4, pp. 658–674, Jul. 2007, doi: 10.1107/S0021889807021206.

[11] K. Cowtan, "Completion of autobuilt protein models using a database of protein fragments," *urn:issn:0907-4449*, vol. 68, no. 4, pp. 328–335, Mar. 2012, doi: 10.1107/S0907444911039655.

[12] P. Emsley, B. Lohkamp, W. G. Scott, and K. Cowtan, "Features and development of Coot," *Acta Crystallogr D Biol Crystallogr*, vol. 66, no. Pt 4, pp. 486–501, 2010, doi: 10.1107/S0907444910007493.

[13] G. N. Murshudov *et al.*, "REFMAC5 for the refinement of macromolecular crystal structures," *urn:issn:0907-4449*, vol. 67, no. 4, pp. 355–367, Mar. 2011, doi: 10.1107/S0907444911001314.

[14] P. D. Adams *et al.*, "PHENIX: a comprehensive Python-based system for macromolecular structure solution," *Acta Crystallogr D Biol Crystallogr*, vol. 66, no. Pt 2, pp. 213–221, 2010, doi: 10.1107/S0907444909052925.

[15] E. F. Pettersen *et al.*, “UCSF Chimera — A Visualization System for Exploratory Research and Analysis,” 2004, doi: 10.1002/jcc.20084.
[16] E. Krissinel and K. Henrick, “Inference of macromolecular assemblies from crystalline state,” *J Mol Biol*, vol. 372, no. 3, pp. 774–797, Sep. 2007, doi: 10.1016/J.JMB.2007.05.022.
[17] G. Kolesov, P. Virnau, M. Kardar, and L. A. Mirny, “Protein knot server: detection of knots in protein structures,” *Nucleic Acids Res*, vol. 35, no. Web Server issue, Jul. 2007, doi: 10.1093/NAR/GKM312.
[18] P. Kumar and D. N. Woolfson, “Socket2: a program for locating, visualizing and analyzing coiled-coil interfaces in protein structures,” *Bioinformatics*, vol. 37, no. 23, pp. 4575–4577, Dec. 2021, doi: 10.1093/BIOINFORMATICS/BTAB631.
[19] S. v. Strelkov and P. Burkhard, “Analysis of α-helical coiled coils with the program TWISTER reveals a structural mechanism for stutter compensation,” *J Struct Biol*, vol. 137, no. 1–2, pp. 54–64, 2002, doi: 10.1006/jsbi.2002.4454.
